# RETRACTION: HIF-1-miR-219-SMC4 Regulatory Pathway Promoting Proliferation and Migration of HCC under Hypoxic Condition

**DOI:** 10.1155/bmri/9798545

**Published:** 2025-06-12

**Authors:** BioMed Research International

**RETRACTION:** C. Yang, F. Huang, L. Deng, X. Yuan, Q. Tao, T. Wang, D. Li, Y. Fan, Q. Peng, and D. Tang. “HIF-1-miR-219-SMC4 regulatory pathway promoting proliferation and migration of HCC under hypoxic condition.” *BioMed Research International,* 2019 (1), 8983704, https://doi.org/10.1155/2019/8983704.

The above article, published online on 7 November 2019 in Wiley Online Library (http://wileyonlinelibrary.com), has been retracted by John Wiley & Sons Ltd. due to concerns identified with Figure 1D and Figure 4E as follows:

- Figure 1D (HCC-LM3-SMC4-NC) and Figure 4E (HCC-LM3/miR-219 inhibitor) appear to be unexpectedly overlapping
- Figure 1D (Huh7-SMC4-siRNA) and Figure 4E (Huh7/Negative control) appear to be appear to be unexpectedly overlapping.

There are also multiple inconsistencies with the western blots throughout the manuscript and the data reported in this article is therefore considered unreliable.

The authors have been informed of this decision but did not provide a response.

